# Carbon Microtube Textile with MoS_2_ Nanosheets Grown on Both Outer and Inner Walls as Multifunctional Interlayer for Lithium–Sulfur Batteries

**DOI:** 10.1002/advs.201903260

**Published:** 2020-09-27

**Authors:** Jiaye Yang, Lihong Yu, Bangbei Zheng, Narui Li, Jingyu Xi, Xinping Qiu

**Affiliations:** ^1^ Institute of Green Chemistry and Energy Tsinghua Shenzhen International Graduate School Tsinghua University Shenzhen 518055 China; ^2^ School of Applied Chemistry and Biological Technology Shenzhen Polytechnic Shenzhen 518055 China; ^3^ Department of Chemistry Tsinghua University Beijing 100084 China

**Keywords:** electrocatalysis, interlayers, lithium–sulfur batteries, polysulfide conversion, synergic effects

## Abstract

The shuttle effect of soluble lithium polysulfides during the charge/discharge process is the key bottleneck hindering the practical application of lithium–sulfur batteries. Herein, a multifunctional interlayer is developed by growing metallic molybdenum disulfide nanosheets on both outer and inner walls of cotton cloth derived carbon microtube textile (MoS_2_@CMT). The hollow structure of CMT provides channels to favor electrolyte penetration, Li^+^ diffusion and restrains polysulfides via physical confinement. The hydrophilic and conductive 1T‐MoS_2_ nanosheets facilitate chemisorption and kinetic behavior of polysulfides. The synergic effect of 1T‐MoS_2_ nanosheets and CMT affords the MoS_2_@CMT interlayer with an efficient trapping‐diffusion‐conversion ability toward polysulfides. Therefore, the cell with the MoS_2_@CMT interlayer exhibits enhanced cycling life (765 mAh g^−1^ after 500 cycles at 0.5 C) and rate performance (974 mAh g^−1^ at 2 C and 740 mAh g^−1^ at 5 C). This study presents a pathway to develop low‐cost multifunctional interlayers for advanced lithium–sulfur batteries.

## Graphical abstract

MoS_2_ nanosheets grown on both outer and inner walls of carbon microtube textile (MoS_2_@CMT) are designed as the interlayer for lithium–sulfur batteries. The synergic effect of MoS_2_ nanosheets and CMT can effectively enhance the diffusion of Li^+^, anchor and catalyze the conversion of lithium polysulfides to Li_2_S_2_/Li_2_S.

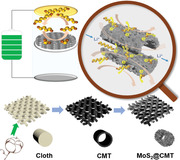

## 1 Introduction

Lithium–sulfur batteries (LSBs), as one of the most promising candidates for next‐generation energy storage, possess high theoretical charge capacity (1672 mAh g^−1^) and energy density (2600 Wh kg^−1^).^[^ ^1^ ^]^ LSBs are highly desirable for a wide range of applications due to environmentally friendly, cost efficiency, and high energy density.^[^ ^2^ ^]^ Nevertheless, the development of LSBs is hindered by low sulfur utilization and sluggish reaction kinetics due to the insulating nature of sulfur and solid reduction products (Li_2_S and Li_2_S_2_).^[^ ^3^, ^4^ ^]^ Furthermore, poorly controlled Li/electrolyte interface^[^ ^5^ ^]^ and volumetric changes during charge/discharge process lead to irreversible structural destruction, mechanical degradation, and rapid capacity decay.^[^ ^6^ ^]^ In addition, the shuttle effect which caused by the formation and transport of various soluble polysulfide intermediates (Li_2_S*_n_*, 4 ≤ *n *≤ 8), resulting in rapid capacity fading, is most desirable to solve.^[^ ^7^ ^]^

Extensive efforts have been concentrated on solving the above obstacles by designing sulfur cathodes,^[^ ^8^ ^]^ optimizing electrolytes,^[^ ^9^ ^]^ and stabilizing Li anodes.^[^ ^10^ ^]^ Although these methods ameliorate the electrochemical performances of LSBs, a working cell still suffers from the shuttle effect. Recently, functional separators/interlayers have been designed to hinder the diffusion of polysulfides and significantly improve the electrochemical performance of LSBs.^[^ ^11^, ^12^, ^13^, ^14^ ^]^ Hence, drawing from large surface area and desirable pore size distribution,^[^ ^15^ ^]^ various types of carbonaceous materials, such as carbon nanotubes (CNTs),^[^ ^16^ ^]^ carbon nanofibers,^[^ ^17^ ^]^ graphene,^[^ ^18^ ^]^ and carbon flakes,^[^ ^19^ ^]^ are used to block the diffusion of polysulfides between anode and cathode. However, weak interactions with polar polysulfide species restrict the role of nonpolar carbonaceous materials.^[^ ^20^ ^]^ Therefore, different types of metal oxides,^[^ ^21^ ^]^ nitrides,^[^ ^22^ ^]^ phosphides,^[^ ^23^ ^]^ and sulfides^[^ ^24^ ^]^ are combined with aforementioned carbonaceous materials to enhance the affinity with polysulfides and simultaneously convert them to Li_2_S_2_/Li_2_S.^[^ ^25^, ^26^ ^]^ Nevertheless, most of these materials own a low electrical conductivity, which means that the immobilized polysulfides remaining on the surface of these materials cannot be completely utilized.^[^ ^27^ ^]^

As 2D transition‐metal dichalcogenides, MoS_2_ is a desirable modified material for LSBs due to its large size S^2−^ anions, which bind to polysulfides easily.^[^ ^28^ ^]^ MoS_2_ nanosheets exhibit either 1T‐MoS_2_ (metallic phase) or 2H‐MoS_2_ (semiconductor phase) structure, where edge sites play a critical role in the conversion of polysulfides.^[^ ^29^ ^]^ Compared with 2H‐MoS_2_, 1T‐MoS_2_ phase possesses active center in edge sites and base planes, exhibits hydrophilicity, and demonstrates 10^7^ times higher conductivity.^[^ ^30^ ^]^ Recently, several research groups have successfully combined MoS_2_ with carbonaceous materials, such as MoS_2_@CNT,^[^ ^31^ ^]^ MoS_2−_ *_x_*/rGO,^[^ ^32^ ^]^ and MoS_2_/graphene,^[^ ^33^ ^]^ to exploit the synergistic effect of MoS_2_ and carbonaceous materials. Nonetheless, there are some disadvantages in these MoS_2_/nanocarbon composites. On one hand, the CNT, rGO and graphene powder‐derived 3D conductive networks are not easy to scale up, which will increase the cost of the LSBs. On the other hand, the utilization of vacuum‐filtration method will results in restacking and agglomeration of MoS_2_ nanosheets, lowering the active surface area and hindering the exposed active sites of MoS_2_ for trapping and conversion of polysulfides.^[^ ^27^ ^]^ Therefore, rational design a scalable free‐standing MoS_2_/carbonaceous hierarchical interlayer is essential for boosting the trapping and conversion ability of polysulfides in practical LSBs.

**Figure 1:**
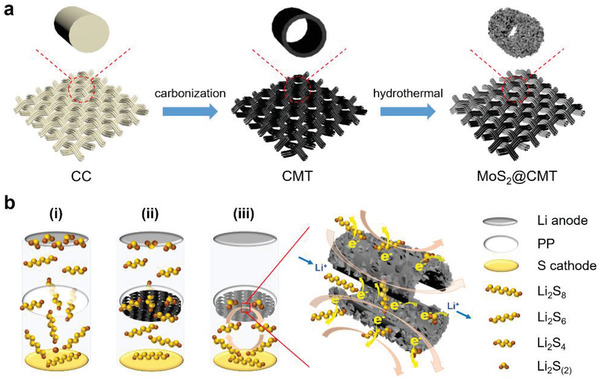
a) Fabrication process of MoS_2_@CMT. b) Schematic illustration of LSBs with various configurations: i) Polypropylene (PP) separator suffers from serious shuttling of polysulfides, ii) CMT interlayer can physically block part of polysulfides, and iii) MoS_2_@CMT interlayer enabling efficient trapping‐conversion of polysulfides.

In our previous work, we have demonstrated that a robust and scalable carbon microtube textile (CMT) can be used as an efficient free‐standing interlayer for LSBs.^[^ ^34^ ^]^ As shown in **Figure** **1**a, the CMT is fabricated by carbonization of commercial cotton cloth (CC), resulting in an extremely low cost of $1 per m^2^, which is beneficial for large‐scale application. However, the CMT interlayer can only block part of the polysulfides in a working LSB because of its nonpolar feature (ii in Figure 1b). To further boost the performance of CMT, we herein report a hierarchical MoS_2_@CMT as multifunctional interlayer for LSBs in which 1T‐MoS_2_ nanosheets are uniformly grown on both outer and inner walls of CMT by a one‐pot hydrothermal method (Figure 1a). In MoS_2_@CMT interlayer based LSB (iii in Figure 1b), the dense decorated 1T‐MoS_2_ nanosheets without significant restacking, which expose more electrochemically active surface area, facilitating the chemisorption and catalytic conversion of polysulfides. The hollow structure of CMT provides channels, favoring electrolyte penetration, Li^+^ diffusion and restrains polysulfides via physical confinement. The synergic effect of 1T‐MoS_2_ nanosheets and CMT functionalize the MoS_2_@CMT interlayer with an efficient trapping‐diffusion‐conversion ability toward polysulfides, leading to significantly enhanced cycling stability and rate capability of the LSBs. Consequently, the MoS_2_@CMT‐based LSBs render initial discharge capacity of 1162 mAh g^−1^ at 0.5 C, and a high specific capacity of 765 mAh g^−1^ preserved after 500 cycles at 0.5 C, indicating a capacity decay rate of 0.068% per cycle. Further, the presence of MoS_2_@CMT interlayer demonstrates high specific capacities of 974 and 740 mAh g^−1^ at 2 and 5 C, respectively, which is 5.3 and 5.7 times higher than the specific capacity of LSB without MoS_2_@CMT interlayer. Besides, the MoS_2_@CMT‐based LSBs achieve a high specific capacity of 1244 mAh g^−1^ with a sulfur loading of 2 mg cm^−2^ at 0.1 C. The current work presents a scalable route to fabricate multifunctional interlayers with low cost for LSBs.

## 2 Results and Discussion

Photographs of CC, CMT, and MoS_2_@CMT depict the macromorphology evolution (Figure S1a, Supporting Information). CC exhibits an apparent shrinkage after carbonization, whereas a small change is observed after the hydrothermal process. Furthermore, excellent flexibility is rendered by CMT and MoS_2_@CMT (Figure S1b,c, Supporting Information). Scanning electron microscope (SEM) images show that the woven structure of CC maintains after carbonization and subsequent hydrothermal process (Figure S2, Supporting Information). In addition, the cross‐sectional SEM images confirm the structural change from the solid cotton fibers to the hollow carbon microtube fibers. The outer and inner walls of hollow CMT fibers (Figure S3, Supporting Information) can provide abundant space for the growth of MoS_2_ nanosheets during the hydrothermal process, resulting in a hierarchical MoS_2_@CMT interlayer (Figure S2c, Supporting Information) with superior trapping/conversion ability toward polysulfides in working Li–S batteries.

**Figure 2:**
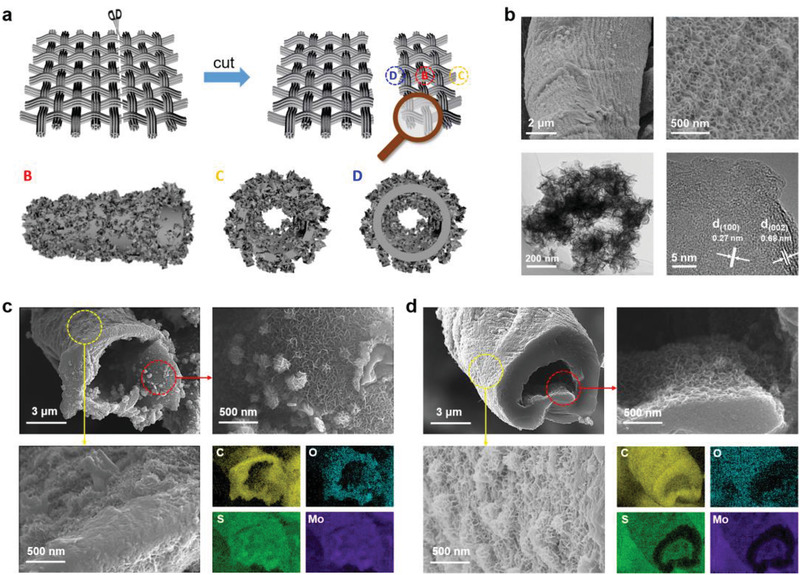
Morphology characterization of MoS_2_@CMT. a) Schematic illustration of various locations (B, C, and D) and corresponding structures for SEM observation, b) SEM, TEM, and HR‐TEM images at location B, c) SEM images and elemental mapping images at location C (original cross‐section of microtube), and d) SEM images and elemental mapping images at location D (intermediate cross‐section of microtube).

**Figure** **2** displays the micromorphology of MoS_2_@CMT. In order to clearly illustrate the growth position of MoS_2_, Figure 2a schematically shows the distribution of MoS_2_ at different locations of CMT, including the top surface (location B), the original cross‐section (location C), and the intermediate cross‐section (location D). We first observed the surface to graph of MoS_2_@CMT (Figure 2b). SEM images demonstrate that MoS_2_ nanosheets are uniformly deposited on the surface of CMT. The higher magnification of SEM image depicts that the thickness of MoS_2_ nanosheets is ≈10 nm (Figure S4, Supporting Information). The homogeneously distributed MoS_2_ nanosheets expose the edge sites without any significant restacking, which implies that MoS_2_@CMT renders high active surface area. Transmission electron microscope (TEM) image further confirms the nanosheet morphology of MoS_2_. High‐resolution TEM (HR‐TEM) image presents a honeycomb arrangement of the atoms with a lattice spacing of 0.27 nm, corresponding to (100) planes of MoS_2_.^[^ ^35^ ^]^ The interlayer distance of 0.68 nm is related to (002) lattice planes of MoS_2_, which indicates the expansion of the interlayer spacing of MoS_2_ nanosheets on the CMT.^[^ ^36^ ^]^ SEM images of the original cross‐section (location C) and intermediate cross‐section (location D) of MoS_2_@CMT are represented in Figure 2c,d, respectively, verifying the uniform growth of MoS_2_ nanosheets on both walls of hollow carbon microtubes. The elemental mapping images validate the homogeneous distribution of C, O, S, and Mo elements in the MoS_2_@CMT and distinguish the original and intermediate cross‐sections. As for the intermediate cross‐section, it was created by cutting the as‐prepared MoS_2_@CMT (Figure 2a). Therefore, we can observe that S and Mo show element rings, while the C and O are concentrated in the middle region, which corroborates the fact that MoS_2_ nanosheets grow on both outside and internal surfaces of hollow carbon microtubes. The SEM results clearly show that even if the length of the carbon microtubes exceeds several hundred microns (Figures S2 and S3, Supporting Information), the MoS_2_ nanosheets can be uniformly grown on the inner/outer walls by a simple hydrothermal method. This can be attributed to the excellent hydrophilicity^[^ ^34^ ^]^ of the carbon microtubes and the larger tube diameter (3–8 µm).

**Figure 3:**
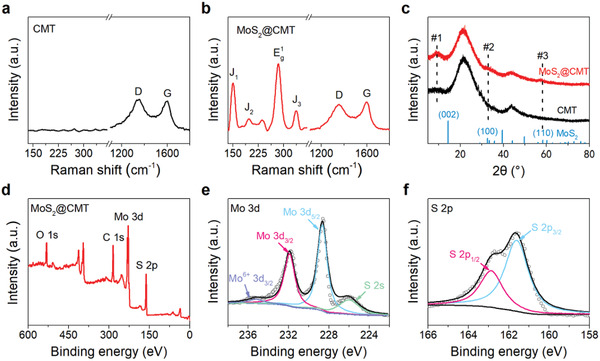
Characterizations of CMT and MoS_2_@CMT. Raman spectra of a) CMT and b) MoS_2_@CMT. c) XRD patterns of CMT and MoS_2_@CMT and d) XPS survey spectra of MoS_2_@CMT. The corresponding high‐resolution XPS spectra of e) Mo 3d and f) S 2p of MoS_2_@CMT.

We then perform Raman spectroscopy to investigate the structure of MoS_2_ and the degree of disorder of carbon microtubes in CMT and MoS_2_@CMT. The Raman spectrum of CMT shows two characteristic peaks at ≈1358 and ≈1594 cm^−1^ (**Figure** **3**a), corresponding to the D and G bands of carbon, respectively.^[^ ^37^ ^]^ There are no obvious peaks before 400 cm^−1^, indicating the purity of CMT. As for MoS_2_@CMT, the characteristic Raman peaks reflect the presence of MoS_2_ (<400 cm^−1^) and CMT (>1300 cm^−1^) (Figure 3b). The Raman peaks at 150 (J_1_), 220 (J_2_), 280 (E_1g_), and 330 (J_3_) cm^−1^ are assigned to the characteristic features of 1T phase of MoS_2_.^[^ ^38^ ^]^ Furthermore, the *I*_D_/*I*_G_ ratio decreases from 1.06 to 0.92 after the deposition of MoS_2_ nanosheets, indicating the loss of the ordered structure of carbon microtubes after the hydrothermal reaction.^[^ ^38^ ^]^ Figure 3c presents X‐ray diffraction (XRD) patterns of CMT and MoS_2_@CMT, where the broad diffraction peaks, located at 2*θ* = 21.4° and 43.8°, correspond to (002) and (100) planes of graphitized carbon of carbon microtubes, respectively.^[^ ^39^ ^]^ The diffraction peaks, located at 2*θ* = 32.7° and 58.4°, represent (100) and (110) planes of MoS_2_ (Powder Diffraction File, No. 37‐1492). An additional peak observed at 7.3° refers to the layer separation of 0.55–0.60 nm, which confirms the absence of restacking in MoS_2_ nanosheets.^[^ ^40^ ^]^ The low‐angle diffraction peak (<10°) is the most convincing identification characteristic of 1T‐MoS_2_.^[^ ^41^ ^]^

The surface composition of MoS_2_@CMT was investigated by X‐ray photoelectron spectroscopy (XPS). Figure 3d shows the XPS survey spectra, which confirm the existence of C, O, S, and Mo elements in MoS_2_@CMT. The high‐resolution Mo 3d spectrum consists of four distinct peaks, as displayed in Figure 3e. The peaks at 231.8 and 228.7 eV can be assigned to Mo 3d_3/2_ and Mo3d_5/2_ of Mo^4+^ in MoS_2_,^[^ ^33^ ^]^ whereas the peak at 235.4 eV can be attributed to 3d_3/2_ of Mo^6+^, indicating the formation of Mo—O—C bond between CMT and MoS_2_ nanosheets.^[^ ^38^ ^]^ A small peak at 225.9 eV can be ascribed to S 2s of MoS_2_, suggesting the formation of Mo‐S bindings.^[^ ^42^ ^]^ Figure 3f presents the high‐resolution XPS spectrum of S 2p, which can be deconvoluted into S 2p_1/2_ and S 2p_3/2_, located at 162.9 and 161.8 eV, respectively, suggesting the S^2−^ state of S element.^[^ ^43^ ^]^ The high‐resolution C 1s and O 1s XPS spectra are illustrated in Figure S5 in the Supporting Information. C 1s spectrum can be fitted into three peaks at 284.6, 285.4, and 286.5 eV, corresponding to C—C, C—O, and C=O, respectively (Figure S5a, Supporting Information). The oxygen‐containing functional groups restrict the movement of polysulfides during charge/discharge process and thus hinder the shuttle effect.^[^ ^19^ ^]^

In order to explore the enhanced properties of MoS_2_@CMT interlayer preliminary, measurements of electrolyte wettability, electric conductivity, and Li_2_S_6_ adsorption capability are performed. In the measurement of electrolyte contact angle (Figure S6, Supporting Information), MoS_2_@CMT exhibits 0° at 0.01 s, showing the excellent wettability of MoS_2_@CMT, which is highly desirable for electrolyte penetration and Li‐ions transportation. In the resistance measurement (Figure S7, Supporting Information), the resistance of MoS_2_@CMT is slightly lower than CMT (30.7 Ω vs 32.3 Ω) due to the direct growth of highly conductive 1T‐MoS_2_. The improved electrical conductivity of MoS_2_@CMT can promote electronic transport and facilitate electrochemical reactions. The Li_2_S_6_ adsorption test is carried out to evaluate the polysulfides adsorption capability of MoS_2_@CMT. Both CMT and MoS_2_@CMT are immersed into Li_2_S_6_ solution for 3 h and the optical images are captured after 0, 1, and 3 h (Figure S8, Supporting Information). After 3 h, the solution with MoS_2_@CMT becomes nearly transparent, whereas the counterpart maintains the initial yellow color. Hence, MoS_2_@CMT possesses a strong physical adsorption ability to anchor polysulfides. The MoS_2_@CMT reacted with Li_2_S_6_ is measured with XPS (Figure S9, Supporting Information). A new peak at 161.5 eV represents Li_2_S_2_.^[^ ^44^ ^]^The peaks at 162.9 and 165.1 eV are assigned to polysulfides.^[^ ^44^, ^45^ ^]^ And the appearance of peaks in the range 168.2–170.9 eV can be attributed to S—O bond in the oxidized sulfur species such as polythionate and sulfate, which derived from the catalytic reaction between MoS_2_@CMT and polysulfides.^[^ ^46^ ^]^ The results prove the strong chemical interaction of MoS_2_@CMT with polysulfides, demonstrating its ability of chemisorption.

**Figure 4:**
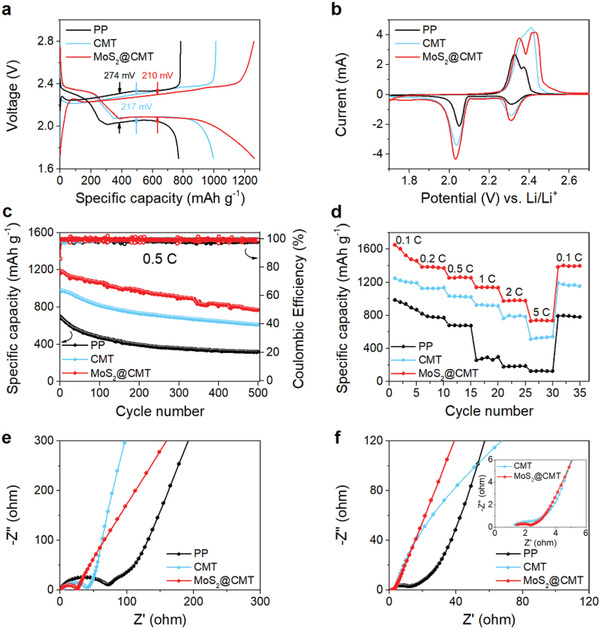
Electrochemical performance of LSBs with CMT and MoS_2_@CMT interlayers. a) Charge/discharge profiles at 0.5 C, b) CV curves at a scan rate of 0.1 mV s^−1^, c) cycling stability at 0.5 C for 500 cycles, and d) rate performance at various current densities. Nyquist plots of cells e) before cycling and f) after 500 cycles at 0.5 C.

Based on excellent electrolyte wettability, lower electrical resistance, and enhanced polysulfide trapping ability of MoS_2_@CMT, we compare the electrochemical performance of LSBs with PP separator, CMT interlayer, and MoS_2_@CMT interlayer. **Figure** **4**a shows the cyclic voltammetry (CV) curves of three LSBs, demonstrating two oxidation peaks and two reduction peaks at around 2.3 and 2.05 V, corresponding to the reduction from S_8_ to soluble long‐chain lithium polysulfides (Li_2_S*_n_*, 4 ≤ *n *≤ 8) and the subsequent reduction from long‐chain lithium polysulfides to Li_2_S_2_/Li_2_S, respectively.^[^ ^47^ ^]^ In addition, MoS_2_@CMT‐based LSB displays stronger redox peaks, corresponding to improved electrochemical reaction kinetics.^[^ ^48^ ^]^ The galvanostatic charge–discharge profiles are consistent with the CV profiles, presenting two discharge plateaus at around 2.3 and 2.05 V. MoS_2_@CMT‐based LSB exhibits an overpotential of 210 mV, smaller than CMT‐ (217 mV) and PP‐based LSBs (274 mV), which is ascribed to the synergistic effect of MoS_2_ and CMT promoting the reaction kinetics and rendering superior electrochemical performance.^[^ ^49^ ^]^ Figure S10 in the Supporting Information demonstrates the charge/discharge profiles of CMT and MoS_2_@CMT without sulfur loading. CMT shows a discharge capacity of 5.7 mAh g^−1^ in the initial cycle. Although MoS_2_@CMT has a relatively high capacity of 7.3 mAh g^−1^ in the initial cycle, it is extremely low and can be ignored in comparison with the MoS_2_@CMT‐based LSB. The results further confirm that the capacity improvement of the S electrode with the MoS_2_@CMT interlayer is due to the adsorption and electrocatalysis of MoS_2_ on LiPSs rather than the capacity contribution from MoS_2_. Figure 4c presents the long‐term cycling performance of PP‐, CMT‐, and MoS_2_@CMT‐based LSBs at 0.5 C. The presence of MoS_2_@CMT interlayer ensures a stable coulombic efficiency of ≈100%, maximum initial discharge specific capacity of 1162 mAh g^−1^ and capacity retention of 92% after 100 charge/discharge cycles. It should be noted that the capacity decay rate of MoS_2_@CMT‐based LSB is only 0.085% per cycle during the first 100 cycles, which is lower than PP‐, CMT‐based LSBs and previously published reports (Table S1, Supporting Information). After 500 cycles at 0.5 C, a high specific capacity of 765 mAh g^−1^ preserved, indicating a capacity decay rate of 0.068% per cycle. Even at the high current densities of 2 and 3 C, MoS_2_@CMT‐based LSB delivers superior cyclic performance (Figure S11, Supporting Information). Figure 4d shows the rate performance of the PP‐, CMT‐, and MoS_2_@CMT‐based LSBs, and the corresponding charge–discharge profiles are illustrated in Figure S12 in the Supporting Information. A higher initial specific capacity of 1645 mAh g^−1^ is rendered by MoS_2_@CMT‐based LSB at 0.1 C, whereas the CMT‐ and PP‐based LSBs exhibit lower initial specific capacities of 1245 and 991 mAh g^−1^, respectively. When the current density gradually increases to 0.2, 0.5, 1, 2, and 5 C, the discharge capacity of MoS_2_@CMT‐based LSB decreases to 1384, 1257, 1136, 974, and 740 mAh g^−1^, respectively. However, a significantly high specific capacity of 1396 mAh g^−1^ is recovered when the current density returns to 0.1 C, indicating that the presence of MoS_2_@CMT interlayer significantly enhances the immobilization of polysulfides. On the contrary, CMT‐ and PP‐based LSBs exhibit severe capacity decay and dull reaction kinetics at high current densities, delivering a specific capacity of 516 and 128 mAh g^−1^ at 5 C, respectively. When the current density returns to 0.1 C, CMT‐ and PP‐based LSBs deliver a low specific capacity of 1193 and 794 mAh g^−1^.

In order to further confirm the practicality of MoS_2_@CMT interlayer, we investigate the rate performance and cyclability with high sulfur loading. The rate performance with a sulfur loading of 2 mg cm^−2^ is shown in Figure S13 in the Supporting Information. Although higher sulfur loading reduces the overall specific capacity of MoS_2_@CMT‐based LSB, the electrochemical performance is still much better than CMT‐ and PP‐based LSBs. Even at a high current density of 2 C, MoS_2_@CMT‐based LSB delivers a high specific capacity of 742 mAh g^−1^, whereas CMT‐ and PP‐based LSBs render a specific capacity of 509 and 168 mAh g^−1^. The cyclability with a sulfur loading of 4.5 mg cm^−2^ is shown in Figure S14 in the Supporting Information. MoS_2_@CMT‐based LSB goes through the initial process of gradual increase in capacity, which is due to the activation of cathode. After ten cycles, MoS_2_@CMT‐based LSB reaches a capacity of 759 mAh g^−1^ and retains 93% of the capacity after 80 cycles. The results further show that MoS_2_@CMT interlayer can achieve adsorption and rapid conversion of polysulfides, thus lead to the good cycling stability under high sulfur mass loading.

Furthermore, the electrochemical impedance spectroscopy (EIS) of PP‐, CMT‐, and MoS_2_@CMT‐based LSBs is carried out to investigate the reaction kinetics and the corresponding Nyquist plots are given in Figure 4e. All Nyquist plots consist of a semicircle in high‐frequency region and a sloping line in low‐frequency region. The semicircle corresponds to the charge transfer resistance (*R*_ct_), whereas the sloping line represents the Warburg impedance (*Z*_w_) related to the Li‐ion diffusion within the sulfur cathode.^[^ ^50^ ^]^ MoS_2_@CMT‐, CMT‐, and PP‐based LSBs exhibit *R*_ct_ values of 23.01, 34.60, and 67.98 Ω, respectively. The smaller value of *R*_ct_ represents the efficient immobilization of polysulfides by MoS_2_@CMT interlayer due to strong chemical absorption, contributing to the lower impedance and superior electrochemical stability. The *R*_ct_ values demonstrate a downward trend after 500 charge/discharge cycles at 0.5 C. Similarly, MoS_2_@CMT‐based LSB remains a smaller *R*_ct_ value (1.63 Ω) than CMT‐ and PP‐based LSBs. The EIS results are attributed to the formation of a stable interface, efficient charge transfer and fast electrochemical reactivity of MoS_2_@CMT interlayer.

MoS_2_@CMT functions as an ideal interlayer material, ensuring efficient diffusion of Li‐ions. CV and Randles–Sevcik equation are utilized to calculate the Li‐ion diffusion coefficient (*D*_Li+_) (Figure S15, Supporting Information).^[^ ^51^ ^]^ The slope of the linear plot of peak current (*i*_p_) versus the square root of the scan rate (*v* ^0.5^) initially reflects the difference in *D*_Li+_ of PP separator, CMT interlayer and MoS_2_@CMT interlayer (Table S2, Supporting Information). The higher slope of MoS_2_@CMT interlayer indicates the fastest Li^+^ transport because of the direct growth of MoS_2_ nanosheets on CMT substrate, avoiding the restacking or aggregation of MoS_2_ nanosheets. In addition, the hollow structure of MoS_2_@CMT will also provide the Li^+^ conduction channels (Figure 1).

**Figure 5:**
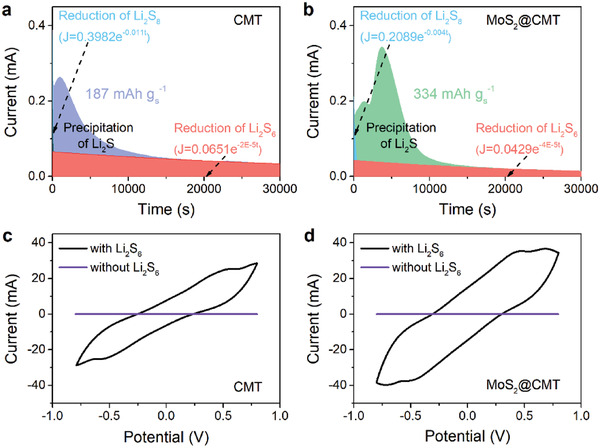
Elucidating the catalytic effect of MoS_2_@CMT. a,b) Potentiostatic discharge curves of a Li_2_S_8_/tetraglyme solution at 2.05 V on different surfaces. The blue/red colors indicate the reduction of Li_2_S_8_/Li_2_S_6_, with the other two colors representing the precipitation of Li_2_S. The capacity of Li_2_S deposition is shown. c,d) CV curves of symmetric cells with identical electrodes in electrolytes with and without Li_2_S_6_ at 0.1 mV s^−1^.

In addition to the efficient Li^+^ diffusion capability, MoS_2_@CMT tackles the barrier of shuttle effect due to excellent polysulfide adsorption capability and catalytic conversion. To further elaborate the catalytic mechanism of MoS_2_@CMT, the Li_2_S precipitation on the surface of CMT and MoS_2_@CMT is investigated by using the Faraday's law,^[^ ^52^ ^]^ as shown in **Figure** **5**a,b. The Li_2_S nucleation capacity on MoS_2_@CMT surface is 334 mAh g^−1^, which is much higher than the CMT surface (187 mAh g^−1^), demonstrating that MoS_2_@CMT significantly accelerates the precipitation of Li_2_S. Figure 5c,d present the CV curves of symmetric cells with identical electrodes of CMT or MoS_2_@CMT, measured in the voltage range of −0.8 to 0.8 V. The response current is increased with the addition of Li_2_S_6_ in the electrolyte. Moreover, MoS_2_@CMT‐based cell affords a higher increase in response current than CMT‐based cell, indicating the enhanced kinetics of the redox reaction of Li_2_S_6_.

Furthermore, PP‐, CMT‐, and MoS_2_@CMT‐based LSBs are disassembled after 500 charge/discharge cycles at 0.5 C, and SEM analysis is carried out to observe the microstructural changes (Figure S16, Supporting Information). The cathode from PP‐based LSB demonstrates large craters and holes, originating from the large volumetric changes of S during the charge/discharge process. However, the cathode from CMT‐based LSB contains a relatively lower number of craters and holes. In the case of the cathode from MoS_2_@CMT‐based LSB, a smooth surface is observed, which does not contain any craters and holes. Hence, SEM analysis provides the visual evidence of the role of MoS_2_@CMT interlayer, which effectively enhanced the utilization of polysulfides by accelerating the chemical adsorption and catalytic process. This can be further confirmed by comparing the colors of the used PP separator in different LSBs (Figure S16d, Supporting Information). The PP separators from PP‐ and CMT‐based LSBs display yellowish color, while the PP separator from MoS_2_@CMT‐based LSB retained almost white surface, demonstrating the effective trapping/conversion ability of MoS_2_@CMT interlayer toward polysulfides.

## 3 Conclusion

In summary, we developed a low‐cost and scalable multifunctional interlayer by growing 1T‐MoS_2_ nanosheets on the outer and inner walls of carbonized cotton‐derived CMT. Such well‐designed MoS_2_@CMT interlayer makes full use of the synergic effect of 1T‐MoS_2_ nanosheets and CMT, and exhibits advantages toward high‐energy density lithium–sulfur batteries. First, the electronically conductive CMT substrate achieves the physical blocking of polysulfides and acts as an upper current collector. Second, the hollow microtube structure of CMT can enhance the diffusion of Li^+^ and facilitate the penetration of electrolyte. Finally, 1T‐MoS_2_ nanosheets with excellent hydrophilicity and conductivity are uniformly distributed without significant restacking, thereby exposing more electrochemically active surface area for adsorption and conversion of polysulfides. Benefiting from the above merits, the lithium–sulfur batteries with the MoS_2_@CMT interlayer render enhanced capacity, cyclic stability and excellent rate performance. Furthermore, a high specific capacity can be realized with a sulfur loading of 2 mg cm^−2^. This work provides an efficient strategy for low‐cost lithium–sulfur batteries.

## 4 Experimental Section

##### Fabrication of CMT and MoS_2_@CMT

The CMT was fabricated by carbonizing the commercial cotton cloth at 950 °C for 2 h. The carbonization was carried out at a heating rate of 5 °C min^−1^ in a tube furnace under argon atmosphere. The MoS_2_@CMT was prepared by using a one‐pot hydrothermal method. Briefly, 0.48 g of glucose, 0.75 g of Na_2_MoO_4_·2H_2_O, and 1.5 g of NH_2_CSNH_2_ were added in 150 mL of deionized (DI) water under magnetic stirring for 15 min. Then, 1 mL of concentrated hydrochloric acid was added into the abovementioned solution and stirred for 5 min. Then, the solution and five pieces of CMT (3 cm × 4 cm) were transferred into a 200 mL Teflon‐lined autoclave and held at 200 °C for 22 h. The resulting MoS_2_@CMT was washed with deionized water several times and dried in a vacuum oven at 60 °C for 24 h.

##### Physical Characterization

The structural analysis was carried out by using XRD (Bruker D8), equipped with Cu‐*K*_*α*_ radiation. The morphology was observed by using SEM (ZEISS SUPRA 55), equipped with an energy dispersive X‐ray spectrometer (EDS, Oxford INCA EDS) and TEM (FEI Tecnai G2 spirit). Raman spectroscopy was carried out by using a Raman spectrometer (Horiba LabRAM HR800). The XPS (Thermo Fisher ESCALAB 250Xi) was used to obtain the chemical composition and valence states of different elements.

##### Adsorption Properties of Polysulfides

A Li_2_S_6_ solution (2.5 mol L^−1^ [S]) was used as the electrolyte and prepared by combining lithium sulfide and sulfur powder with a molar ratio 5:1 in 1 m lithium bis(trifluoromethane sulfonyl)imide (LiTFSI) and 1 wt% LiNO_3_ in 1,3‐dioxolane (DOL) and dimethoxymethane (DME) solution (1:1 by volume) under vigorous magnetic stirring at 50 °C for 24 h. Five pieces of CMT or MoS_2_@CMT circular discs with a diameter of 19 mm were added into lithium polysulfide stock solution containing 20 µL Li_2_S_6_ solution and 10 mL DOL and DME solution (1:1 by volume).

##### Electrochemical Measurements

The electrochemical cells were assembled by using the following procedure: 60 wt% sulfur, 30 wt% Super P, and 10 wt% polyvinylidene fluoride (PVDF) binder were dried at 60 °C in a vacuum oven for 2.5 h and, then, homogenized in *N*‐methyl‐2‐pyrrolidone by continuous stirring for 5 h to obtain a uniform slurry. The as‐prepared slurry was coated on a carbon‐coated aluminum foil and dried at 55 °C for 12 h in a vacuum oven. The cathodes were obtained by punching the slurry‐coated aluminum foil into circular pieces with a diameter of 12 mm. The areal mass loading of the cathode was ≈2.0 mg cm^−2^. On the other hand, pure lithium foil and Celgard 2400 separator were used as the anode and separator, respectively. The interlayer‐containing LSBs were assembled by inserting CMT or MoS_2_@CMT interlayers (with a diameter of 19 mm) between the cathode and separator. 1 m LiTFSI and 1 wt% LiNO_3_ in DOL and DME solution (1:1 by volume) was used as the electrolyte. The interlayer‐free LSBs were assembled by adding 20 µL of the electrolyte in cathode side and anode side, separately. And interlayer‐containing LSBs were assembled by adding 60 µL of the electrolyte in cathode side and 20 µL in the anode side.

CV was performed by using a GAMRY electrochemistry workstation (Interface 5000E) in the voltage range of 1.7–2.8 V at a scan rate of 0.1 mV s^−1^. EIS was carried out in the frequency range of 100 kHz to 10 mHz. The battery testing system (Neware, CT‐4008‐5V10mA) was used to assess the galvanostatic charge/discharge, cyclic performance, and rate capability at room temperature (25 ± 2 °C) in the voltage window of 1.7–2.8 V.

##### Nucleation of Lithium Sulfide

A Li_2_S_8_ solution (0.2 mol L^−1^) was prepared by combining lithium sulfide and sulfur powder with a molar ratio 7:1 in tetraglyme under vigorous magnetic stirring at 50 °C for 24 h, which has been used as the electrolyte. The CMT and MoS_2_@CMT, with a diameter of 12 mm, were used as working electrodes and lithium foil was used as a counter and reference electrode. In the cell assembly process, 20 µL of Li_2_S_8_‐free electrolyte was dropped on the lithium anode and 25 µL of Li_2_S_8_‐containing electrolyte (0.2 mol L^−1^) was dropped on the cathode. The cell was galvanostatically discharged to 2.06 V under current of 0.112 mA and, then, the potential was maintained at 2.05 V until the current dropped below 10^−5^ A. Driven by an overpotential of 0.01 V, Li_2_S was deposited on the heterostructure surface. Based on Faraday's law, the energy was gathered to evaluate the nucleation/growth rate of lithium sulfide on the heterostructure surface.

##### Symmetrical Cell Fabrication and Characterization

A Li_2_S_6_ solution (2.5 mol L^−1^ [S]) was prepared in the above lithium polysulfides adsorption measurement. The CMT and MoS_2_@CMT electrodes, with a diameter of 12 mm, were used to construct the symmetric cells, where 20 µL of Li_2_S_6_‐containing electrolyte was dropped on both electrodes. CV was carried out at a scan rate of 10 mV s^−1^ in the voltage range of −0.8 to 0.8 V by using the GAMRY electrochemical workstation (Interface 5000E).

## Conflict of Interest

The authors declare no conflict of interest.

## Supporting information

Supporting Information
