# Supplementary material for: Carbon Microtube Textile with MoS2 Nanosheets Grown on Both Outer and Inner Walls as Multifunctional Interlayer for Lithium–Sulfur Batteries
Source: Adv Sci (Weinh). 2020 Sep 27;7(21):1903260. doi: 10.1002/advs.201903260 (PMC7610341; doi:10.1002/advs.201903260)
Supplement: Supplementary file 1 — Supporting Information [file ADVS-7-1903260-s001.pdf]

© 2020 Wiley-VCH GmbH

ADVANCED SCIENCE Open Access

Supporting Information

for *Adv. Sci.*, DOI: 10.1002/advs.201903260

Carbon Microtube Textile with MoS_2 Nanosheets Grown on Both Outer and Inner Walls as Multifunctional Interlayer for Lithium-Sulfur Batteries

Jiaye Yang, Lihong Yu, Bangbei Zheng, Narui Li, Jingyu Xi and Xinping Qiu

This file includes:
Figure S1-S16, Table S1-S2 and references.

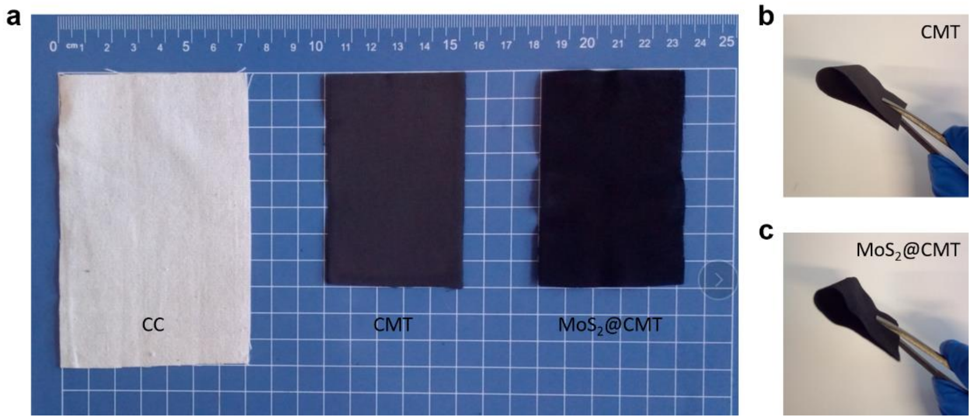

Figure S1. Macro-morphology evolution. (a) Photograph of CC, CMT and MoS_2@CMT, (b-c) flexible feature of CMT and MoS_2@CMT.

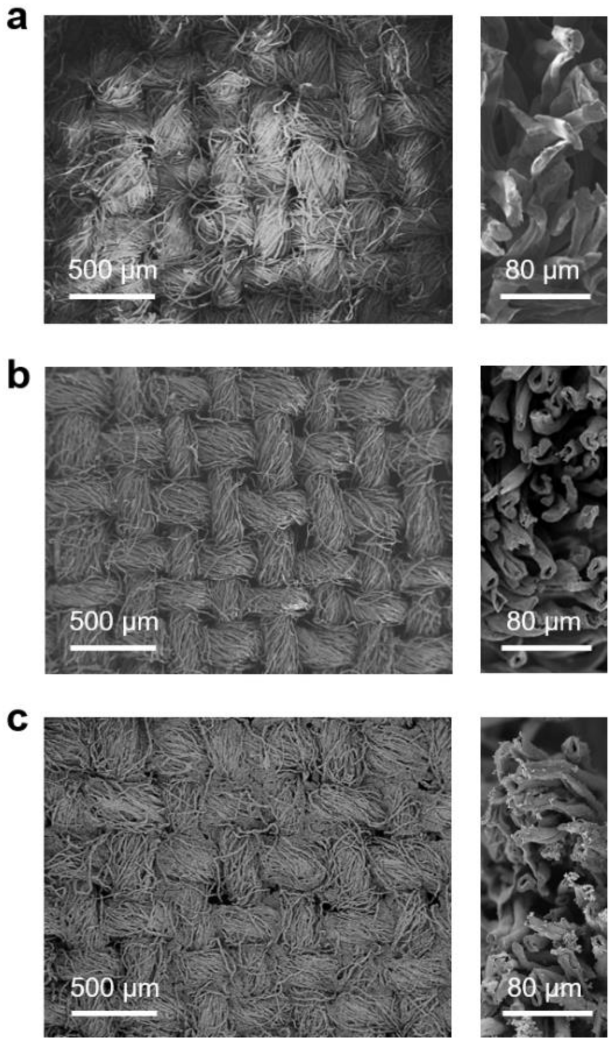

Figure S2. Micro-morphology evolution. Surface (left) and cross-sectional (right) SEM images of (a) CC, (b) CMT, and (c) MoS_2@CMT.

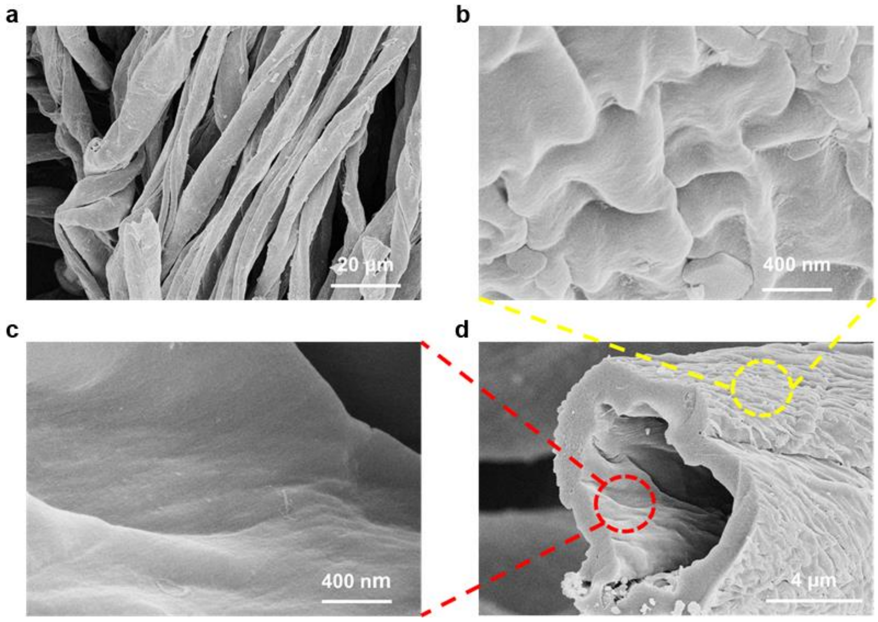

Figure S3. SEM images of CMT. (a) Low- magnification, (b) outside surface, (c) internal surface, (d) cross-section.

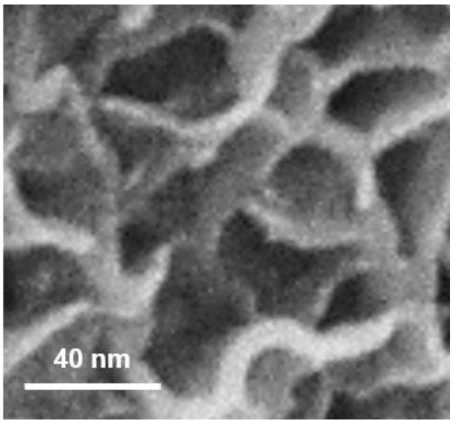

Figure S4. SEM image of MoS_2@CMT with high magnification.

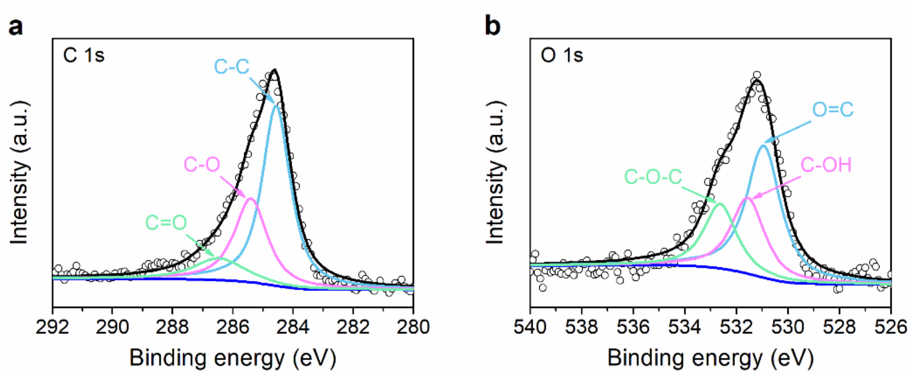

Figure S5. High-resolution XPS spectrum of MoS_2@CMT. (a) C 1s, (b) O 1s.

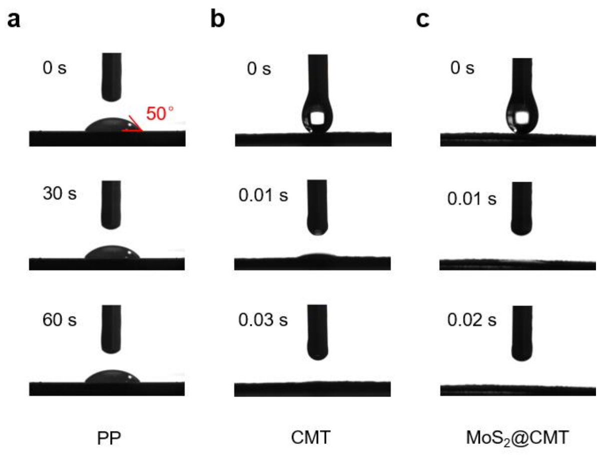

Figure S6. Electrolyte contact angle of (a) PP, (b) CMT, and (c) MoS_2@CMT.

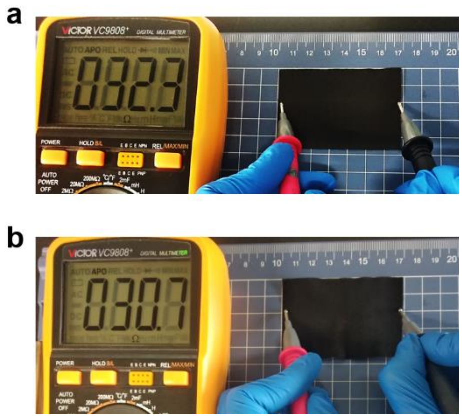

Figure S7. Photographs of the resistance measurement of (a) CMT and (b) MoS_2@CMT.

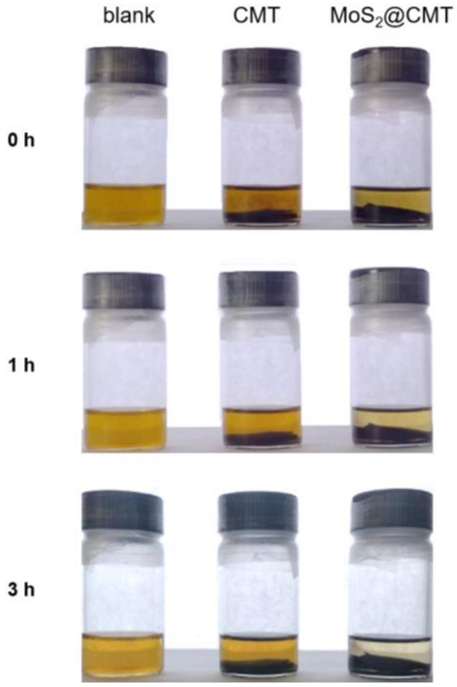

Figure S8. The images of the adsorption capability of CMT and MoS_2@CMT towards Li_2S_6.

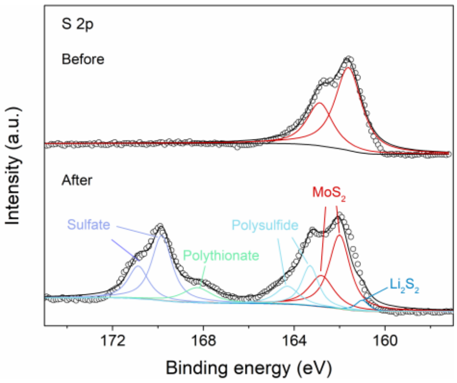

Figure S9. Comparison of the S 2p XPS peaks of MoS_2@CMT before and after Li_2S_6 adsorption.

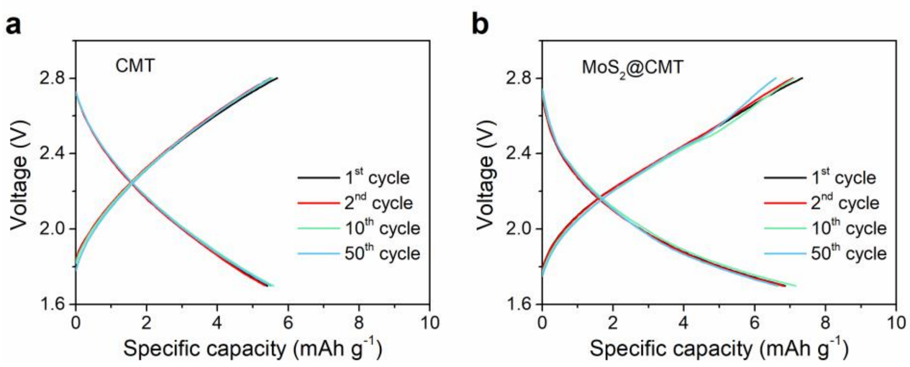

Figure S10. Charge/discharge profiles of (a) CMT and (b) MoS_2@CMT without sulfur loading.

Pure lithium foil and Celgard 2400 separator were used as the anode and separator, respectively. The CMT or MoS_2@CMT interlayer (with a diameter of 19 mm) was used as the cathode. 1 M LiTFSI and 1 wt. % $LiNO_3$ in DOL and DME solution (1:1 by volume) was used as the electrolyte. The volume of the electrolyte used in each battery was 80 μL. Galvanostatic charge/discharge was carried out in the voltage window of 1.7-2.8 V at a current density of 0.5 C (corresponding to the current of LSB with 1 mg cm^{-2} of S loading).

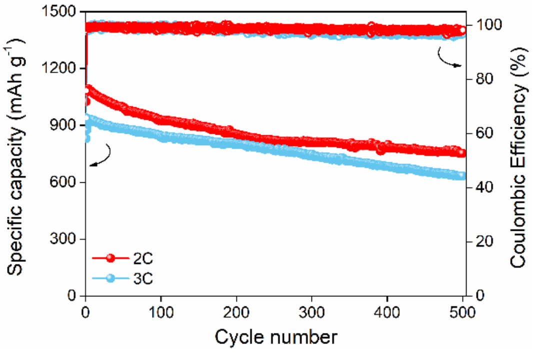

Figure S11. The cyclability of the Li-S batteries with MoS_2@CMT interlayer at 2 and 3 C rates.

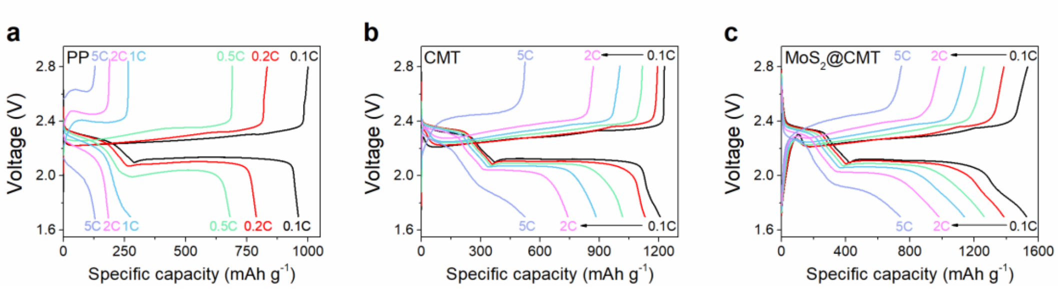

Figure S12. Charge/discharge profiles of the Li-S batteries employing (a) PP separator, (b) CMT interlayer, and (c) MoS_2@CMT interlayer at different rates.

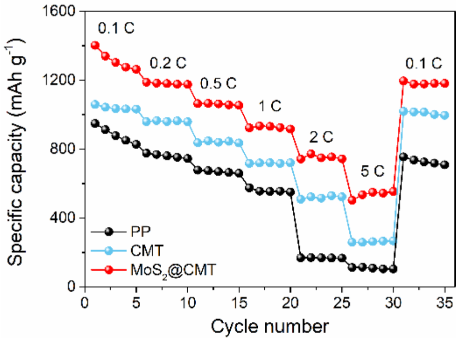

Figure S13. The rate performances of PP separator, CMT interlayer and MoS_2@CMT interlayer under the sulfur loading of 2 mg cm^{-2}.

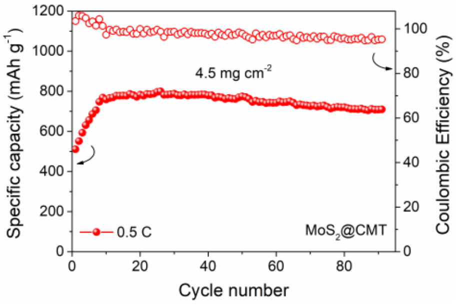

Figure S14. The cyclability of the Li-S battery with MoS_2@CMT interlayer at 0.5 C with a high sulfur loading of 4.5 mg cm^{-2}.

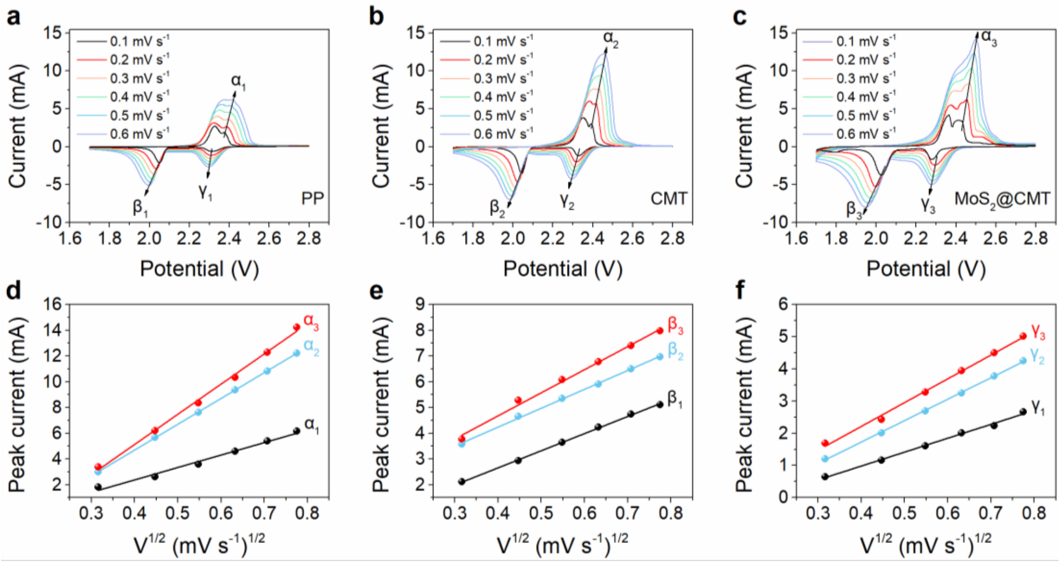

Figure S15. Cyclic voltammetry curves and the linear relationship of the peak current (i_p) vs. the square root of the scan rate ($v^{1/2}$) for PP separator (a, d), CMT interlayer (b, e) and MoS_2@CMT (c, f) interlayer at various scanning rates between 1.7 and 2.8 V.

The peak current (I_p) and the square root of the scan rate ($v^{0.5}$) exhibit a linear relationship, which reflects a diffusion-controlled process. PP-, CMT- and MoS_2@CMT-based LSBs are tested under the same conditions for comparison. The anodic peak (α) refers to the reaction from Li_2S_2/Li_2S to high-order Li_2S_x (4≤x≤8) and, finally, to S_8. On the other hand, the cathodic peak (γ), located at ~2.35 V, is related to the reduction from S_8 to soluble Li_2S_x (4≤x≤8). The other cathodic peak (β) is located at ~2.05 V, which is associated with a further reduction of high-order polysulfide to Li_2S_2/Li_2S.

Table S1. The comparison of electrochemical performance and relevant parameters of this work with other similar reports in the literatures about the interlayers at 0.5C rate for 100 cycles.

Composition	Sulfur loading ($mg\ cm^{-2}$)	Sulfur content (%)	Remaining capacity ($mAh\ g^{-1}$)	Capacity decay per cycle (%)	Ref.
MoS_2@NC	1.2	60	880.0	0.196	[1]
3D MoS_2	1.2	60	941.0	0.144	[2]
PEO/PAA	1.0	60	806.0	0.232	[3]
SiO_2@MoS_2	1.2	60	876.0	0.193	[4]
NCF	1.2	70	902.8	0.197	[5]
CNFO@CNT	1.0	60	869.0	0.223	[6]
CCC	1.0	60	818.5	0.153	[7]
MoS_2@CMT	1.0	60	1093.4	0.085	This work

Table S2. Li^+ diffusion coefficients (D_{Li+}) of batteries with different configurations calculated from the Randles-Sevcik equation.

Sample	D_{Li+} at peak α [$cm^2\ s^{-1}$]	D_{Li+} at peak β [$cm^2\ s^{-1}$]	D_{Li+} at peak γ [$cm^2\ s^{-1}$]
PP	1.26×10^{-10}	5.99×10^{-11}	2.52×10^{-11}
CMT	5.43×10^{-10}	7.29×10^{-11}	6.00×10^{-11}
MoS_2@CMT	7.44×10^{-10}	1.09×10^{-10}	7.39×10^{-11}

Li^+ diffusion coefficient (D_{Li+}):

D_{Li+} was calculated through the Randles-Sevcik equation:

$$i_p = 2.69 \times 10^5 n^{3/2} A D^{1/2} v^{1/2} \Delta C_0$$

in which i_p is the peak current (mA), n is the number of electrons transferred in the reaction (for Li-S batteries, n=2), A is the electrode area (cm^2), D is the Li^+ diffusion coefficient ($cm^2\ s^{-1}$), ΔC_0 is the change of Li^+ concentration ($mol\ cm^{-3}$), and v is the scan rate.

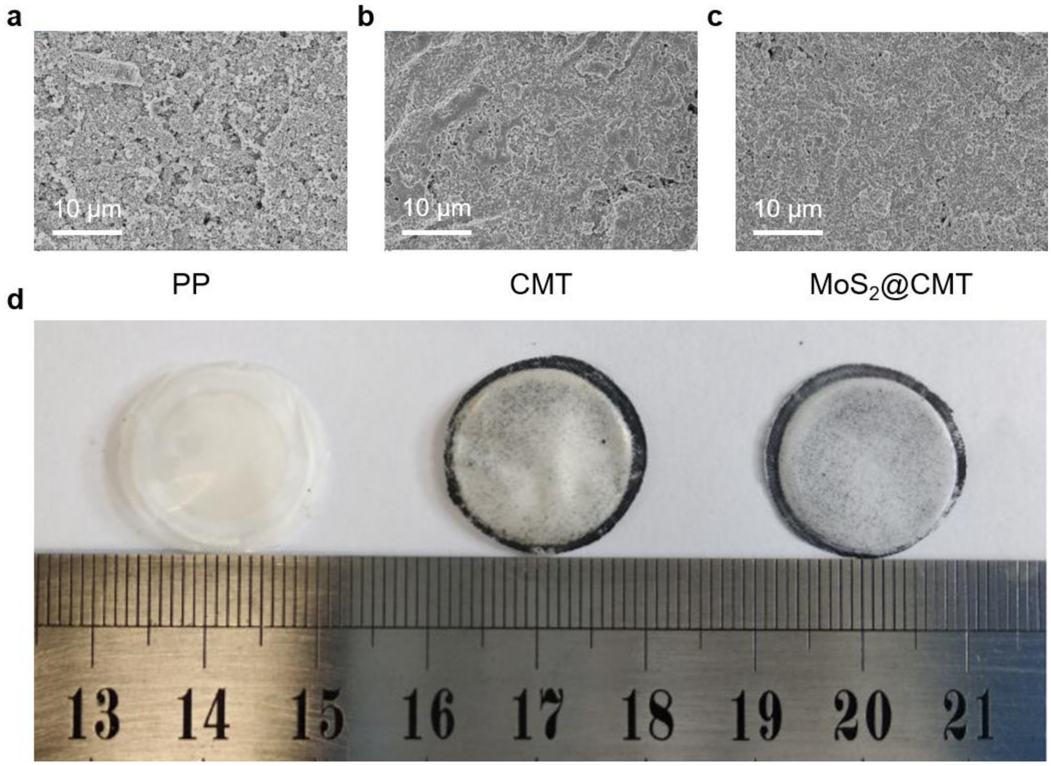

Figure S16. The analysis of the batteries after cycling at 0.5 C for 500 cycles. Surface SEM images of the cathodes removed from the Li-S batteries using: (a) PP separator, (b) CMT interlayer, and (c) MoS_2@CMT interlayer. (d) Photograph of the corresponding PP separators.

References

[1] J. Y. Wu, X. W. Li, H. X. Zeng, Y. Xue, F. Y. Chen, Z. G. Xue, Y. S. Ye, X. L. Xie, *J. Mater. Chem. A* **2019**, *7*, 7897.

[2] J. Y. Wu, H. X. Zeng, X. W. Li, H. J. Pei, Z. G. Xue, Y. S. Ye, X. L. Xie, *ACS Appl. Energy. Mater* **2019**, *2*, 1702.

[3] E. T. Kim, J. Park, C. Kim, A. G. Simmonds, Y. E. Sung, J. Pyun, K. Char, *ACS Macro. Lett.* **2016**, *5*, 471.

[4] J. Y. Wu, N. You, X. W. Li, H. X. Zeng, S. Li, Z. G. Xue, Y. S. Ye, X. L. Xie, *J. Mater. Chem. A* **2019**, *7*, 7644.

[5] Z. X. Cao, J. Zhang, Y. M. Ding, Y. L. Li, M. J. Shi, H. Y. Yue, Y. Qiao, Y. H. Yin, S. T. Yang, *J. Mater. Chem. A* **2016**, *4*, 8636.

[6] T. Liu, S. M. Sun, J. L. Hao, W. Song, Q. H. Niu, X. L. Sun, Y. Wu, D. P. Song, J. F. Wu, *ACS Appl. Mater. Interfaces* **2019**, *11*, 15607.

[7] B. B. Zheng, N. R. Li, J. Y. Yang, J. Y. Xi, *Chem. Commun*. **2019**, *55*, 2289.
